# Lung directed antibody gene transfer confers protection against SARS-CoV-2 infection

**DOI:** 10.1136/thoraxjnl-2021-217650

**Published:** 2022-02-14

**Authors:** Yue Du, Kamran M Miah, Omar Habib, Helena Meyer-Berg, Catriona C Conway, Mariana A Viegas, Rebecca Dean, Dwiantari Satyapertiwi, Jincun Zhao, Yanqun Wang, Nigel J Temperton, Toby P E Gamlen, Deborah R Gill, Stephen C Hyde

**Affiliations:** 1 NDCLS, Radcliffe Department of Medicine, University of Oxford, Oxford, UK; 2 National Clinical Research Center for Respiratory Disease, Guangzhou Institute of Respiratory Health, the First Affiliated Hospital of Guangzhou Medical University, Guangzhou Medical University, State Key Laboratory of Respiratory Disease, Guangzhou, Guangdong, China; 3 Medway School of Pharmacy, University of Kent, Canterbury, UK

**Keywords:** COVID-19, respiratory infection, viral infection

## Abstract

**Background:**

The COVID-19 pandemic continues to be a worldwide threat and effective antiviral drugs and vaccines are being developed in a joint global effort. However, some elderly and immune-compromised populations are unable to raise an effective immune response against traditional vaccines.

**Aims:**

We hypothesised that passive immunity engineered by the in vivo expression of anti-SARS-CoV-2 monoclonal antibodies (mAbs), an approach termed vectored-immunoprophylaxis (VIP), could offer sustained protection against COVID-19 in all populations irrespective of their immune status or age.

**Methods:**

We developed three key reagents to evaluate VIP for SARS-CoV-2: (i) we engineered standard laboratory mice to express human ACE2 via rAAV9 in vivo gene transfer, to allow in vivo assessment of SARS-CoV-2 infection, (ii) to simplify in vivo challenge studies, we generated SARS-CoV-2 Spike protein pseudotyped lentiviral vectors as a simple mimic of authentic SARS-CoV-2 that could be used under standard laboratory containment conditions and (iii) we developed in vivo gene transfer vectors to express anti-SARS-CoV-2 mAbs.

**Conclusions:**

A single intranasal dose of rAAV9 or rSIV.F/HN vectors expressing anti-SARS-CoV-2 mAbs significantly reduced SARS-CoV-2 mimic infection in the lower respiratory tract of hACE2-expressing mice. If translated, the VIP approach could potentially offer a highly effective, long-term protection against COVID-19 for highly vulnerable populations; especially immune-deficient/senescent individuals, who fail to respond to conventional SARS-CoV-2 vaccines. The in vivo expression of multiple anti-SARS-CoV-2 mAbs could enhance protection and prevent rapid mutational escape.

**Key messages**

**What is the key question?**

- Can we generate an in vivo model of SARS-CoV-2 infection based on standard laboratory mice, for testing new therapeutics such as passive vaccination with anti-SARS-CoV-2 antibodies?

**What is the bottom line?**

- Using a mimic of SARS-CoV-2 based on recombinant lentivirus pseudotyped with SARS-CoV-2 Spike protein, we created a humanised in vivo mouse model of SARS-CoV-2 infection, and showed long-term, passive vaccination by gene transfer of antibody sequences.

**Why read on?**

- Our humanised mouse model and SARS-CoV-2 mimic offers a rapid, inexpensive and efficient method to evaluate therapeutic interventions to halt SARS-CoV-2 infection. It will be of interest to researchers studying COVID-19 and other respiratory pathogens and, importantly, can be implemented under standard laboratory biosafety conditions without the need to breed and maintain transgenic animals.

## Introduction

The current COVID-19 pandemic, caused by SARS-CoV-2, has ravaged the globe. Many of the vaccine candidates being developed have yielded positive results in clinical trials, generating high levels of antibodies^1 2^ and providing clinical protection.^3^ However, the induction of such protective immunity is entirely dependent on the treated individual’s immune system to develop antigen-specific immunity, and it remains unclear whether diverse populations will respond to the antigen-based vaccine regimens to the same extent. In all likelihood, people will respond to the current vaccines to different degrees and some groups of individuals with poor immunogenicity will struggle to raise a protective immune response.

An alternative strategy is to use vector-mediated immunoprophylaxis (VIP) against SARS-CoV-2 infection, which could circumvent some limitations. VIP involves the delivery of genes encoding neutralising antibodies into target cells via gene transfer; subsequently, the monoclonal antibody (mAb) protein is synthesised in vivo, secreted into the local milieu and ultimately the systemic circulation. Viral vectors can be exploited for VIP, including recombinant Adeno-Associated Virus (rAAV) vectors that provide long-term and stable transgene expression with low vector immunogenicity and high tolerability.^4^ In particular, rAAV-mediated delivery of neutralising antibodies is a promising strategy against HIV,^5^ filovirus,^6^ respiratory syncytial virus^7^ and influenza virus.^8–10^ More recently, recombinant simian immunodeficiency virus (SIV) pseudotyped with the fusion (F) and haemagglutinin-neuraminidase (HN) surface glycoproteins from Sendai virus (rSIV.F/HN)^11^ has also been used to express broadly neutralising mAbs in the airways to protect against a supra-lethal influenza infection.^9^ To our knowledge, there have been no published, peer-reviewed reports on the application of VIP for SARS-CoV-2.

In this study, we delivered the rAAV and rSIV.F/HN gene transfer platforms via intranasal and intramuscular administration routes to express NC0321, a prototypical SARS-CoV-2 neutralising mAb. We then challenged the mAb-treated mice with S-LV (a SARS-CoV-2 pseudovirus created from a recombinant HIV1 lentiviral vector pseudotyped with the D614G derivative of the SARS-CoV-2 Spike (S) protein). We used S-LV infection to investigate the prophylactic efficacy of the NC0321 mAb produced by the in vivo gene transfer vectors. These proof-of-principle studies demonstrate that viral infection can be inhibited by vector-mediated delivery of anti-SARS-CoV-2 mAb genes. We call this strategy ‘*CO*VID-19 *V*ectored *I*mmuno*p*rophylaxis’ (COVIP). Importantly, the COVIP approach could offer potent protection against authentic SARS-CoV-2 infection in populations that fail to respond to conventional SARS-CoV-2 vaccines.

## Materials and methods

Detailed methods can be found in online supplemental information.

10.1136/thoraxjnl-2021-217650.supp1 Supplementary data

## Results

### S-LV as a mimic of SARS-CoV-2

**Figure 1:**
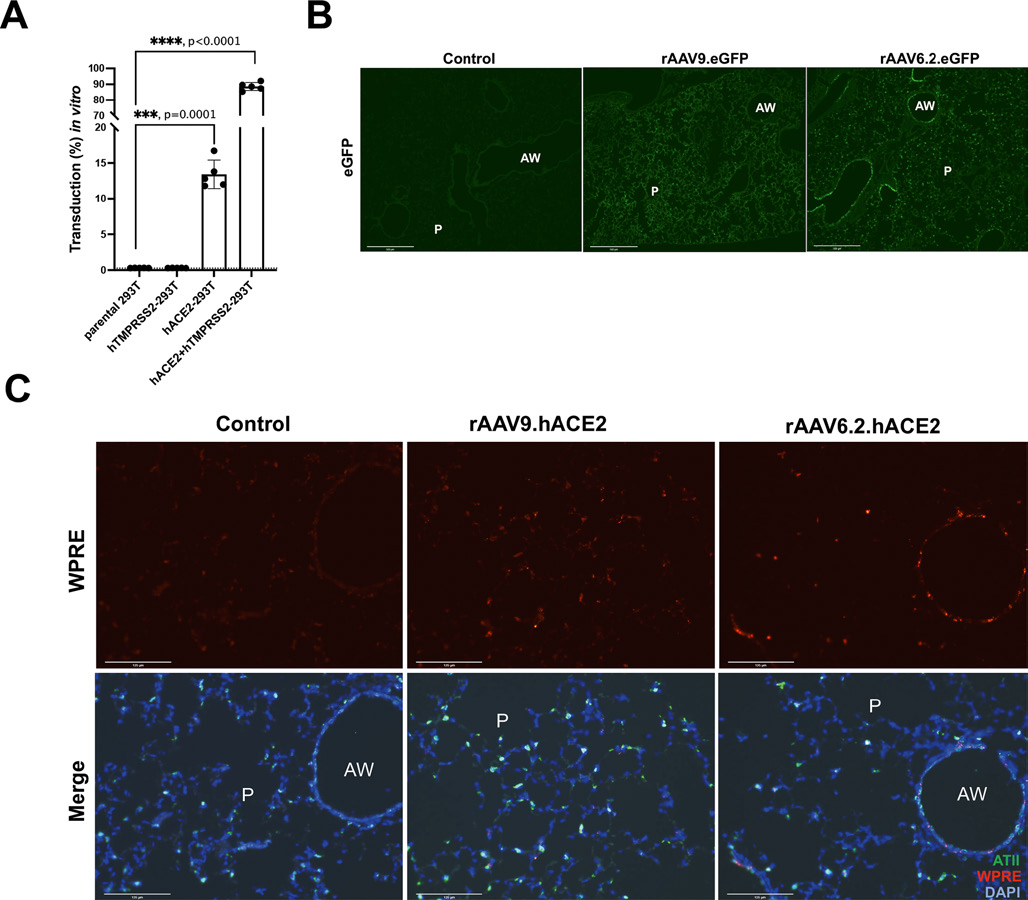
S-LV infection requires hACE2, which can be supplied to mouse lungs by rAAV in vivo gene transfer. (A) WT parental HEK293T/17 cells and HEK293T/17 cells expressing hTMPRSS2, hACE2 or both hACE2 and hTMPRSS2 as indicated, were infected with an S-LV expressing enhanced green fluorescent protein (eGFP). The percentage of S-LV transduced cells was evaluated by flow cytometry. The dotted line represents the limit of quantification. One-way analysis of variance (ANOVA), with Dunnett’s multiple comparisons test (ns and ***** represent p>0.05 and p<0.0001, respectively). (B) Lung immunohistochemistry for eGFP was assessed in BALB/c (9-week-old) mice 14 days after intranasal delivery of 1xD-PBS (control), 1E11 GC of rAAV9 or 7E10 GC rAAV6.2 vectors expressing eGFP (n=3/group). Scale bar=500 μm. (C) Lung sections were subjected to RNAscope in situ hybridisation analysis 14 days after intranasal delivery of 1xD-PBS (control), 1E11 GC of rAAV9 or 7E10 GC rAAV6.2 vectors expressing hACE2 (n=3/group); hACE2 vector-specific WPRE probe (red), alveolar type II cell specific *Sftpb* probe (green), 4′,6-diamidino-2-phenylindole (DAPI) stained nuclei (blue). AW, airway; P, parenchyma. Scale bar=125 μm.

We aimed to generate a non-replicative mimic of SARS-CoV-2 capable of a single-cycle of infection for use under simple laboratory conditions. Importantly, cellular entry of SARS-CoV-2 relies on the viral Spike protein binding to the human angiotensin-converting enzyme 2 (hACE2) receptor, an interaction synergised by cleavage of Spike by transmembrane protease serine 2 (TMPRSS2).^12^ We hypothesised that a third-generation HIV lentiviral vector pseudotyped with the SARS-CoV-2 Spike protein (termed S-LV) would retain similar receptor dependencies. We found that S-LV particles could be readily prepared and showed that, similar to SARS-CoV-2 and other SARS-CoV-2 surrogates,^12^ S-LV infection of cells in vitro was absolutely dependent on hACE2 and was significantly enhanced in the presence of hTMPRSS2 (figure 1A).

### rAAV vector can mediate hACE2 expression in vivo

Since S-LV pseudovirus could mimic SARS-CoV-2 infection in vitro, we next aimed to create an in vivo infection model to evaluate potential therapeutic interventions. However, laboratory mice are not naturally susceptible to COVID-19 infection due to ACE2 receptor incompatibility.^13^ Others have chosen to generate, breed and use hACE2 expressing transgenic mice to overcome this limitation.^14^ As a more accessible alternative, we provided hACE2 and (in some studies) hTMPRSS2 in trans to facilitate SARS-CoV-2 or S-LV entry, thus generating a murine model of SARS-CoV-2 infection.

We used in vivo delivery of both rAAV9 and rAAV6.2 vectors to provide the necessary cellular receptors. Vectors carrying hACE2 or the reporter eGFP were administered to mouse lungs via intranasal instillation and 14 days post-delivery, we observed abundant eGFP expression with both vectors. For rAAV9, eGFP expression was largely restricted to the parenchyma of the lung, predominantly in cells with an alveolar type I (ATI) morphology. In contrast, rAAV6.2 directed eGFP expression in both the lung parenchyma, predominantly in cells with an alveolar type II (ATII) morphology, and in cells of the conducting airway (figure 1B). The significant sequence homology between human and murine (m)ACE2 meant that distinguishing their expression by IHC was challenging,^15^ therefore in situ hybridisation was used to detect vector-derived hACE2 expression via the linked WPRE sequence. figure 1C shows that consistent with the observed eGFP signal, hACE2 expressed from rAAV9 was rarely observed in the conducing airway and was largely restricted to the lung parenchyma, while rAAV6.2 vector expression was observed in cells of the conducting airway, terminal bronchi and alveoli.

### rAAV vector-mediated hACE2 expression facilitates S-LV infection

**Figure 2:**
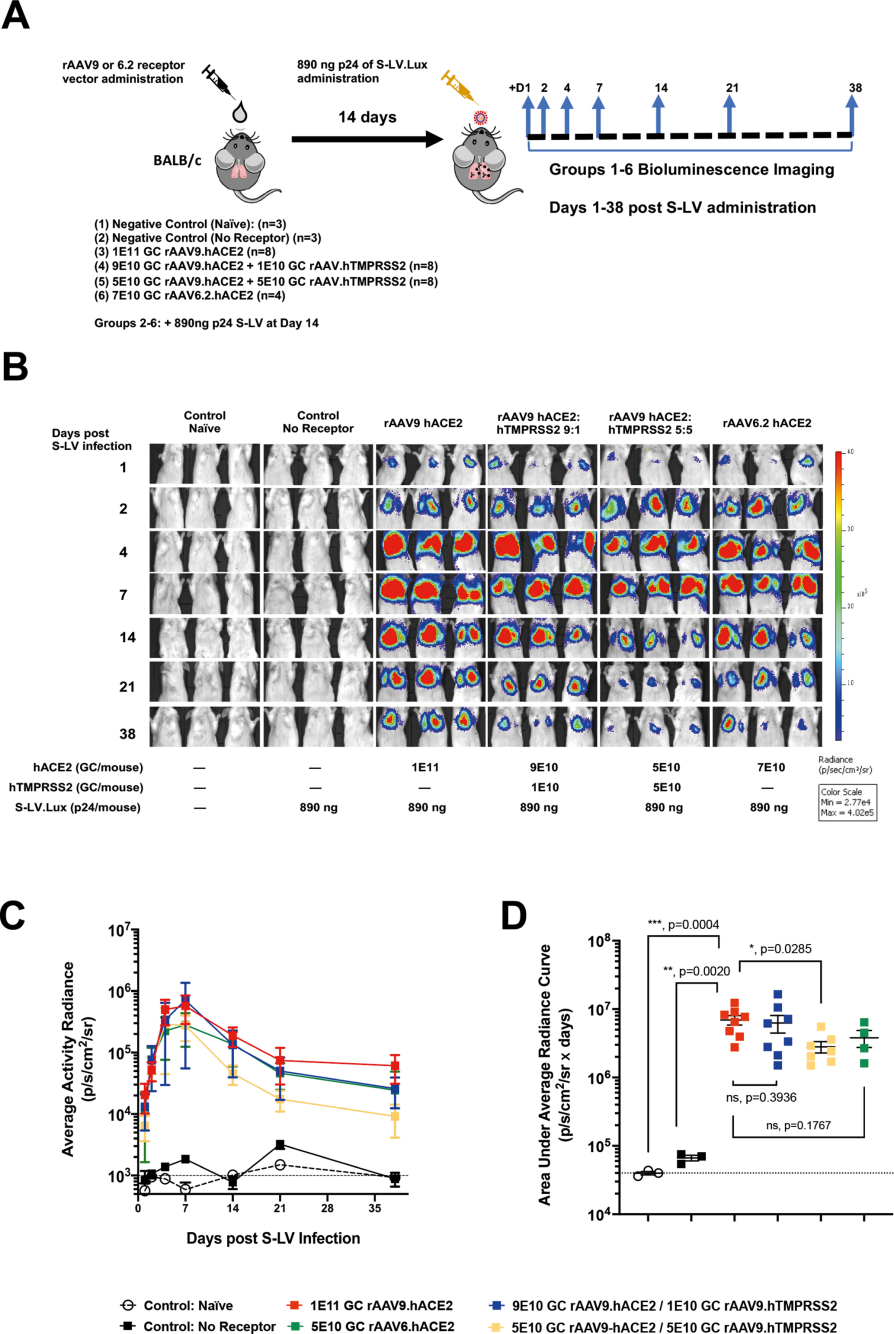
In vivo delivery of hACE2 allows the SARS-CoV-2 mimic S-LV to infect the lungs of standard laboratory mice. (A) Experimental design for in vivo investigation of hACE2/hTMPRSS2 delivery via rAAV vectors to support S-LV infection. BALB/c mice (n=4–8/group) were transduced intranasally with the indicated rAAV vector(s) or vehicle (1xD-PBS). At 14 days post-rAAV delivery, as indicated, lungs were infected with 890 ng p24 of an S-LV expressing firefly luciferase. S-LV dependent in vivo luciferase bioluminescence was monitored for each animal 1, 2, 4, 7, 14, 21 and 38 days post-S-LV infection. (B) Representative in vivo bioluminescence images of mice pretreated 14 days previously with the indicated doses of rAAV.hACE2 and hTMPRSS2 vectors and, as indicated, subsequently challenged with 890 ng p24 of S-LV at day 0. Repeated bioluminescence imaging of S-LV dependent luciferase expression was performed at the indicated time points. Bioluminescence values (photons/s/cm^2^/sr) are presented as a pseudocolour scale as indicated. (C) Time-course of bioluminescence for the indicated treatment groups after infection with 890 ng p24 of S-LV. Symbols represent group mean±SD, n=4–8 per group. The dotted line indicates the mean naïve background signal. (D) Area under curve of bioluminescence values (photons/s/cm^2^/sr×days) for each animal in B and C was computed, symbols represent individual animals and group mean±SD (ANOVA, Dunnett’s multiple comparison against the unlabelled treatment group (1E11 GC rAAV9.hACE2); ns, * and **** represent p>0.05, <0.05 and <0.0001, respectively).

Having established that hACE2 and hTMPRSS2 could be provided in trans to the murine airway via rAAV vectors, we then asked whether hACE2 could facilitate S-LV transduction in murine lungs, and whether infectivity could be enhanced by hTMPRSS2 coexpression. To address this, mice were first treated intranasal with 7E10 genome copies (GC) rAAV6.2 hACE2, 1E11 GC rAAV.hACE2, or cocktails of rAAV9.hACE2 and rAAV9.hTMPRSS2 vectors where the total rAAV9 dose delivered was fixed at 1E11 GC. Mice were infected 14 days later with 890 ng of p24 of S-LV Luciferase (intranasal) and monitored for S-LV-dependent luciferase expression kinetics (figure 2A). Consistent with the in vitro study findings, mouse lungs were refractory to S-LV infection in the absence of hACE2 expression. In contrast, mouse lungs that were primed with hACE2 by either rAAV9 or rAAV6.2 showed abundant luciferase expression after infection with S-LV. Luciferase activity was detectable above background from as early as 24 hours after S-LV infection with signal intensity increasing to a peak at approximately 7 days (figure 2B). Consistent with the lentiviral vector heritage of S-LV, luciferase expression was long-lived, though it should be noted that an early peak of signal intensity (approximately 7 days post-infection) fell to plateau at around 21 days post-infection (figure 2C). The S-LV mediated luciferase expression was monitored over a 38-day time-course, showing luciferase signal intensity achieved with hACE2 priming was more than 200-fold greater than without priming (p<0.0001, figure 2D). While the signal intensity achieved with rAAV6.2.hACE2 priming tended to be ~twofold lower than that achieved with rAAV9 (approximately 100-fold over no priming) this difference failed to reach significance (p=0.2722). Interestingly, and in contrast with our in vitro findings, incorporation of 1E10 GC of rAAV9.hTMPRSS2 had no positive benefit on S-LV infection in vivo and the use of 5E10 rAAV9.hTMPRSS2 significantly reduced (p=0.0221) the S-LV signal to only 60-fold over background (figure 2D). Nevertheless, the higher absolute signal observed using rAAV9.hACE2, together with the substantially lower production yields of rAAV6.2.hACE2 restricting the priming dose that could be delivered, led us to focus on rAAV9.hACE2 priming in all subsequent studies.

### S-LV kinetics and dose-dependency in hACE2-expressing mice

**Figure 3:**
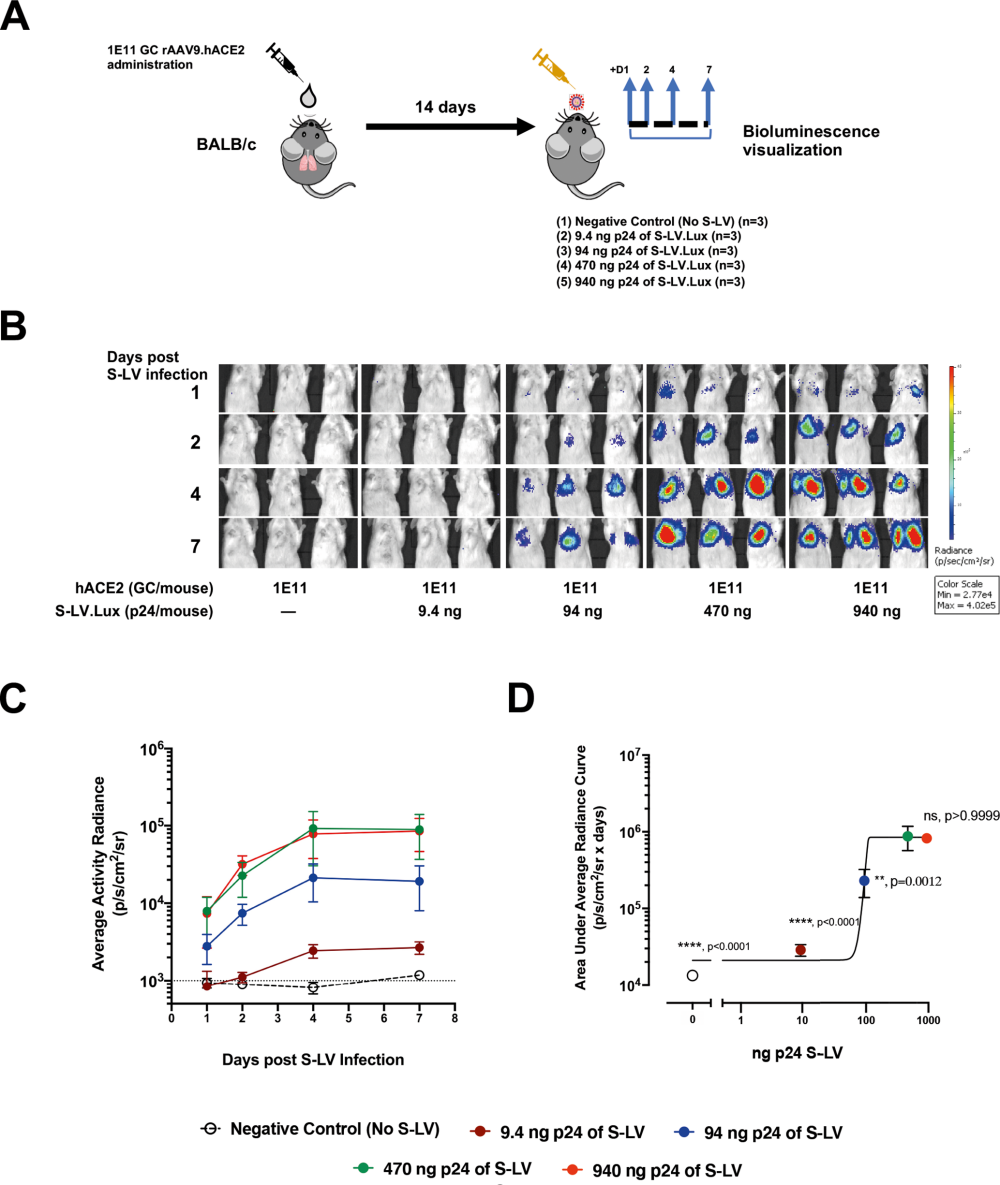
S-LV mouse lung infection is limited by hACE2 availability. (A) Experimental design for in vivo investigation of S-LV dose-response. BALB/c mice (n=3/group) were dosed intranasal with 1E11 GC rAAV9.hACE2 to establish hACE2 expression. Fourteen days later, lungs were infected with 0–940 ng p24 of an S-LV expressing firefly luciferase. S-LV dependent in vivo luciferase bioluminescence was monitored for each animal 1, 2, 4 and 7 days post-S-LV infection. (B) Representative in vivo bioluminescence images of mice, treated as described in (A). Repeated bioluminescence imaging to monitor S-LV dependent luciferase expression was performed at the indicated time points. Bioluminescence values (photons/s/cm^2^/sr) are presented as a pseudocolour scale as indicated. (C) Time-course of bioluminescence after S-LV infection for the indicated treatment groups as described in (A). Symbols represent group mean±SD, n=4–8 per group. The dotted line indicates the mean naïve background signal. (D) Area under curve of bioluminescence (photons/s/cm2/sr×days) for each animal in C) was computed, symbols represent group mean±SD (ANOVA, Dunnett’s multiple comparison against the unlabelled treatment group (470ng p24); ns, **, *** and **** represent p>0.05, p<0.05, <0.001 and <0.0001, respectively).

To gain a more thorough insight into the transduction kinetics of S-LV, we performed dose titration studies in hACE2-expressing mice which were infected with 0, 9.4, 94, 470 and 940 ng p24 of S-LV (figure 3A). We observed a dose-dependent increase in S-LV mediated luciferase expression (figure 3B). As in the previous studies, in vivo bioluminescence in each hACE2-expressing mouse rose to a peak at approximately 7 days post-infection (figure 3C). Notably, infection with either 470 or 940 ng p24 of S-LV produced comparable lung luciferase activity (p>0.9999, figure 3D), consistent with S-LV infection in this model being limited by the rAAV9-mediated hACE2 expression above 470 ng of p24 of S-LV. Importantly, the 470 ng p24 dose of S-LV produced significantly higher lung luciferase activity than the 0, 9.4 or 94 ng p24 dose of S-LV (p<0.001, p<0.001 and p=0.0012, respectively). To avoid any limitation in S-LV signal, we proceeded with the saturating S-LV challenge dose of 470 ng p24 S-LV in subsequent therapeutic protection studies.

### In vitro and in vivo IgG expression mediated by AAV and SIV.F/HN vectors

**Figure 4:**
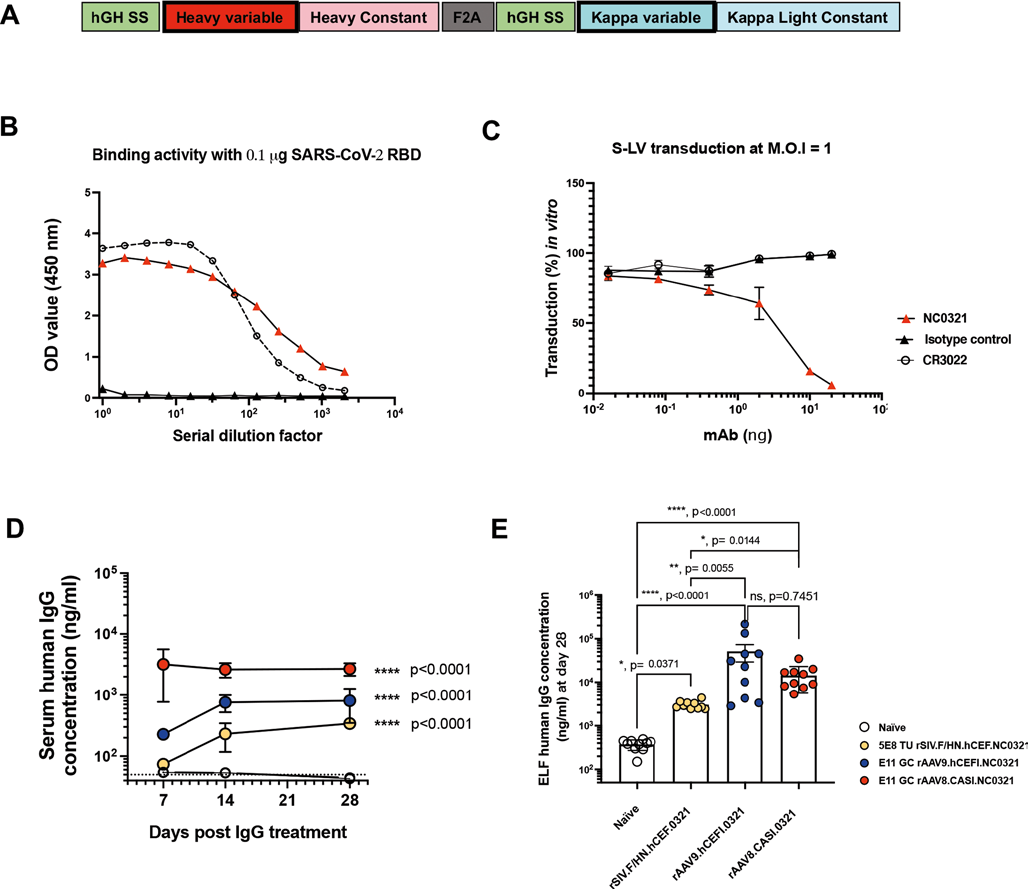
NC0321 mAb expression by rAAV and rSIV.F/HN vectors. (A) Schematic of a codon-optimised, single open-reading frame, human IgG mAb cDNA. Regions encoded include IgG heavy and kappa light chain variable and constant regions, with each proceeded by a human growth hormone signal sequence (hGH SS) and joined by a Furin/2A (F2A) protein cleavage site. (B) The SARS-CoV-2 receptor binding domain (RBD) protein binding activity of the anti-SARS-CoV-2 mAbs NC0321 and CR3022 single open reading frame protein expressed from an rSIV.F/HN vector) was examined by ELISA. Binding activity of OD at 450 nm is proportional to the amount of antibody bound to the SARS-CoV-2 RBD protein. (C) The neutralising activity of anti-SARS-CoV-2 mAb NC0321 (single open reading frame protein expressed from an rSIV.F/HN vector) to block S-LV infection (multiplicity of infection 1) of an hACE2-293T cell line was examined. In (B and C) a single open reading frame anti-influenza mAb T1-3B acted as an isotype negative control; and CR3022 an anti-SARS-CoV-2 neutralising mAb, that can bind but not neutralise SARS-CoV-2 was used as a comparator.^40^ (D) Serum human IgG levels in mice were determined at day 7, 14 and 28 after transduction with the indicated doses of NC0321 expressing vector using ELISA (ANOVA, Dunnett’s multiple comparison against the unlabelled treatment group; *** and **** represent <0.001 and <0.0001, respectively). The levels of human IgG observed in naïve animals is indicated by the dotted line. (E) BALF of mice from (D) was collected at day 28 post-transduction with NC0321 expressing vector, and human IgG levels measured using ELISA; IgG levels in epithelial lining fluid (ELF) were computed by comparison of urea levels in BALF and serum (Kruskal Wallis, Dunn’s multiple comparison against the unlabelled treatment group; ns, **,*** and **** represent p>0.05, <0.01, <0.001 and <0.0001, respectively). Each dot represents an individual mouse and data are presented as endpoint titres (mean±SD).

To establish proof-of-principle for COVIP, we wished to express in vivo a potent anti-SARS-CoV-2 mAb to inhibit S-LV infection in our mouse model. The mAb NC0321 was originally isolated from the convalescent serum of a patient recovered from SARS-CoV-2 infection in China (Zhao JC, in preparation). We established that the single-open reading frame version of NC0321 used (figure 4A and online supplemental figure 1A) could both potently bind the receptor binding domain (RBD) portion of SARS-CoV-2 Spike protein (figure 4B) and block S-LV infection in our in vitro cell model (figure 4C). We then examined the ability of alternative gene transfer vector configurations to mediate expression of NC0321 IgG in vivo. Following delivery of rSIV.F/HN.NC0321 and rAAV9.NC0321 vectors via the intranasal route, and rAAV8.NC0321 via intramuscular injection, we monitored NC0321 expression levels in mouse sera at 7, 14 and 28 days post-delivery. All groups treated with an NC0321 expressing vector contained significantly more NC0321 mAb in the serum than control animals (p<0.0001; figure 4D). Delivery of rAAV8 via the intramuscular route resulted in the most rapid accumulation of NC0321, reaching a peak (~3.9 µg/mL) in serum within 7 days, which was essentially sustained to the end of the time-course (~2.5 µg/mL at day 28). Vectors delivered via the intranasal route had both slower kinetics (reaching a plateau after approximately 14 days post-delivery) and lower, sustained serum levels (~0.8 µg/mL and ~0.3 µg/mL for rAAV9 and rSIV.F/HN, respectively). At the end of the study, 28 days after vector delivery, all mice were culled and BALF collected to determine levels of NC0321 in lung epithelial lining fluid (ELF). All groups treated with vector expressing NC0321 contained significantly more NC0321 mAb in the ELF than control animals (all p<0.05; figure 4E). Delivery via rAAV9 intranasal treatment resulted in the highest NC0321 ELF levels (~65 µg/mL), significantly higher than achieved with rSIV.F/HN, also delivered via the intranasal route (~3 µg/mL; p=0.0055). In contrast, rAAV8 intramuscular delivery was associated with intermediate ELF levels (~18 µg/mL) that were not significantly different (p=0.7451) from those achieved with rAAV9. Taken together, these observations indicate that gene transfer vectors expressing anti-SARS-CoV-2 mAb, delivered via intranasal (rAAV9 or rSIV.F/HN) or intramuscular (for rAAV8) routes, results in abundant serum and ELF accumulation of mAb protein.

### In vivo viral vector-mediated protection against S-LV infection

**Figure 5:**
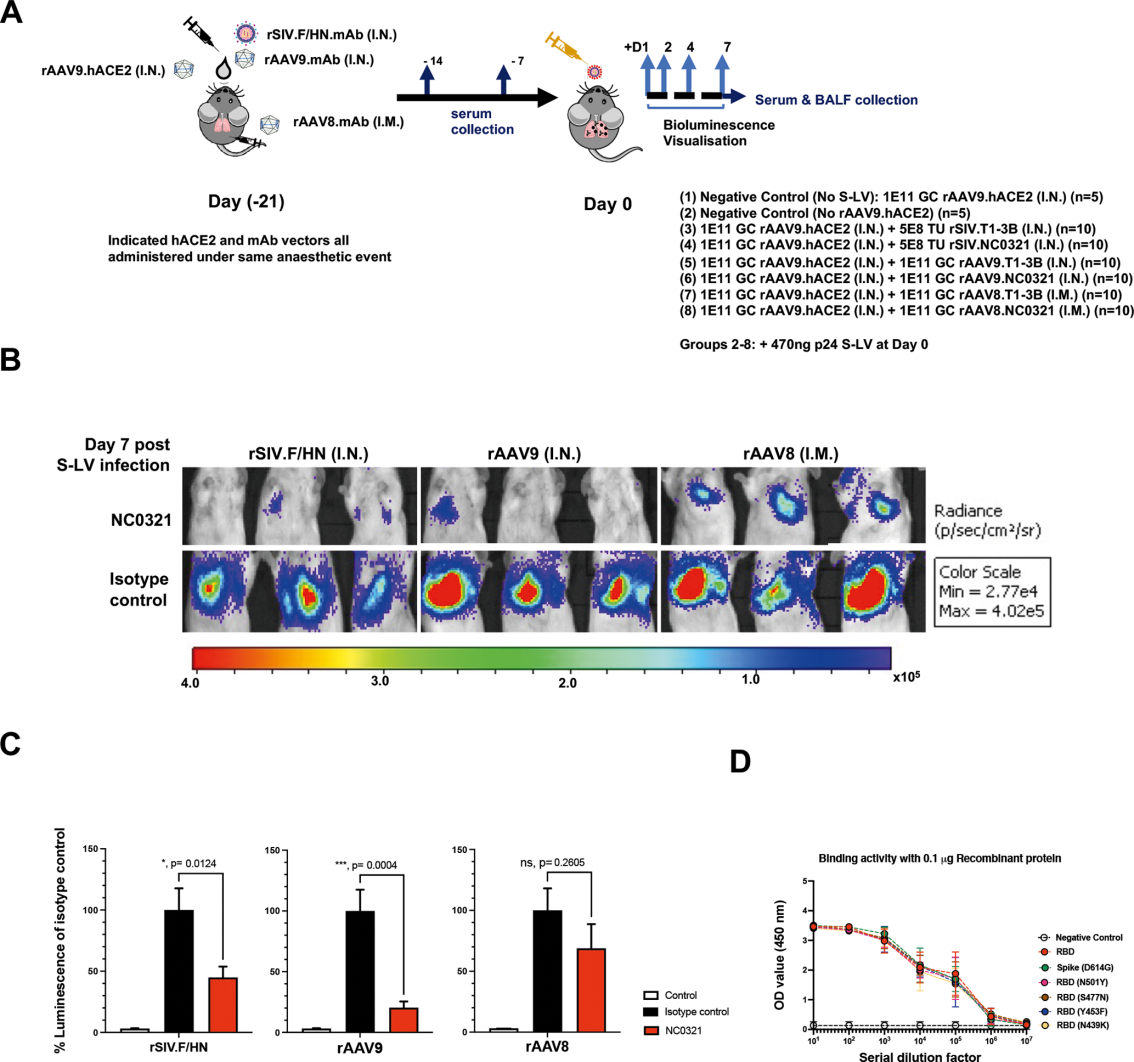
Vector-mediated expression of NCO321 antibody protects against S-LV infection. (A) Experimental design to test efficacy of the *CO*VID-19 *V*ectored *I*mmuno*p*rophylaxis strategy in vivo. Groups of mice were dosed as indicated (groups 1–7) under a single anaesthesia to establish hACE2 and NC0321 expression. Twenty-one days later, animals were infected with an S-LV expressing firefly luciferase (day 0) and, subsequently, S-LV dependent in vivo luciferase bioluminescence monitored for each animal on days 1, 2, 4 and 7. Serum and BALF samples were taken at indicated times for determination of NC0321 IgG levels. (B) Vectored delivery and expression of NC0321 to mouse lungs significantly inhibits S-LV infection. Groups 1–7 of BALB/c mice (n=10/group) were treated as indicated and after 21 days infected with 470 ng p24 of an S-LV expressing firefly luciferase (day 0). Representative images of in vivo bioluminescence are shown 7 days post-S-LV infection for each of the six treatment groups. Bioluminescence values (photons/s/cm^2^/sr) are presented as a pseudocolour scale as indicated. (C) Area under curve of bioluminescence (photons/s/cm^2^/sr×days) for each animal in B was computed. To aid visualisation, bioluminescence values were normalised such that T1-3B isotype control values were 100%. Control values were obtained from animals that were infected with S-LV but had not received rAAV9-hACE2. Group mean±SD is indicated (ANOVA, Dunnett’s multiple comparison against the unlabelled treatment group; ns, **, *** and **** represent p>0.05, <0.01, <0.001 and <0.0001, respectively). (D) Serum from mice 28 days post receiving rAAV8.NC0321 was collected, and limiting dilutions were made to measure the binding activity against SARS-CoV-2 Spike or receptor binding domain proteins of newly emerging SARS-CoV-2 variants as indicated. Negative control is binding activity observed with cell culture medium only.

After confirming NC0321 mAb expression in mouse sera via three different in vivo gene transfer strategies, it was important to determine whether the expressed mAb could reduce S-LV infection. As a control, we utilised gene transfer vectors expressing anti-influenza mAb T1-3B^16^ of the same IgG isotype as NC0321. Twenty-one days after hACE2 and NC0321/T1-3B expression was established in the lungs of BALB/c mice, we infected the study animals from figure 4D, E with 470 ng p24 of S-LV (figure 5A). Luciferase expression mediated by S-LV infection was monitored for 7 days post challenge; in vivo imaging data are presented for representative animals (figure 5B), and all individual mice and treatment groups (online supplemental figure 1B, C). While minor variations in the S-LV signal between the three T1-3B treatment groups were noted, most likely a consequence of the complex study design using three gene transfer vectors (online supplemental figure 1D), these failed to reach significance suggesting no major impact on functional mAb or hACE2 levels. Crucially, over the course of the study (figure 5C), the S-LV luciferase signal intensity achieved on treatment with rAAV9.NC0321 was substantially reduced (~0.71 log or 79.6% protection; p=0.004 compared with T1-3B treatment. Similarly, treatment with rSIV.F/HN NC0321 also significantly reduced the S-LV luciferase signal intensity (~0.25 log or 55.1% protection; p=0.0124). Interestingly, while the S-LV luciferase signal achieved on treatment with rAAV8.NC0321 tended to be lower than that with negative control T1-3B mAb, indicating a modest positive treatment effect (~0.18 log or 31.2% protection), this was not statistically significant (p=0.2605). Together, these data suggest that the intranasal delivery of rSIV.F/HN or rAAV9.NC0321 can confer robust protection against a saturating infectious dose of a SARS-CoV-2 mimic.

Importantly, we also confirmed that NC0321 from mouse serum retained biological function in an in vitro neutralisation assay. As expected, sera from mice receiving the control rAAV8.T1-3B vector showed no neutralising activity against an S-LV expressing eGFP, whereas sera from mice receiving the rAAV8.NC0321 vector demonstrated potent neutralising activity (online supplemental figure 1E); this confirms that rAAV8.NC0321 treatment resulted in the production of biologically active NC0321. Moreover, binding assays showed that rAAV8.NC0321 sera were able to bind to six different RBD proteins including some with mutations that appear to confer enhanced infection or the potential to escape pre-existing immunity.^17^ These include Wuhan strain as reference, S^G614^, RBD^N501Y^, RBD^N439K^, RBD^Y453F^ and RBD^S477N^ mutants (figure 5D).

## Discussion

In this study, we first generated S-LV, a third-generation lentiviral vector pseudotyped with the SARS-CoV-2 Spike protein. Like native SARS-CoV-2, cellular infection by S-LV requires hACE2. Subsequently we created an in vivo model of SARS-CoV-2 infection that can be readily produced using standard laboratory animals, by expressing hACE2 in trans from rAAV hACE2 vectors. Importantly, there appeared to be a positive correlation between the dose of rAAV.hACE2 and the S-LV infection in this humanised mouse model. Our result suggests that rAAV.hACE2 priming should permit authentic SARS-CoV-2 infection. Thus our in-house humanised mouse model, generated by rAAV-mediated hACE2 overexpression, should be suitable for studying authentic SARS-CoV-2 infections and evaluating other prophylactic or therapeutics options. A similar approach to mouse model generation was reported in the studies performed by Israelow *et al*,^18^ Han *et al* 19 and Sun *et al* 20 where both adenoviral vector (rAd) and rAAV vector approaches were used. Importantly, rAAV transduction is associated with lower vector-mediated inflammation and immunogenicity than rAd treatment,^21^ a feature that may allow generation of a more informative mouse model. Importantly, while murine models of SARS-CoV-2 infection tend not to demonstrate the full range of pathology observed following human infection,^22^ they can provide simple, rapid, pharmacodynamic assays to evaluate interventions to modulate viral titres. One major limitation of the hACE2 expressing murine model we adopted is that the biodistribution of hACE2 mediated by intranasal rAAV9 transduction may not be identical to that of natural hACE2 in human lungs—where expression is predominantly noted on the apical surface of alveolar type II cells.^23^ Crucially, studies described here could be completed in standard animal laboratories without the need for the very high levels of biological containment required for utilising SARS-CoV-2, and without the cost and animal wastage associated with the maintenance, breeding and supply of hACE2 transgenic animals. Therefore, this unique rAAV-hACE2/S-LV model could provide a rapid and efficient method to researchers interested in studying COVID-19 and other respiratory pathogens, regardless of the limitations of biosafety level and the availability of commercial humanised animal models.

Subsequently, using our humanised (hACE2-expressing) murine model, we evaluated the performance of alternative VIP strategies. We chose rSIV.F/HN, rAAV9 and rAAV8 vectors to establish NC0321 expression in vivo as we, and others, have had previous positive experiences expressing a range of IgG molecules with these vectors.^9 24–26^ We established a significant degree of protection against infection of 470 ng p24 of our SARS-CoV-2 mimic at, or near, the primary site of inoculation of respiratory pathogens. Both rAAV9 and rSIV.F/HN mediated NC0321 expression via intranasal delivery can result in significant prophylactic efficacy against a saturating infectious inoculum of S-LV. It would be reasonable to assume that this treatment effect would be even more marked against a more typical environmentally acquired (sub-saturating) SARS-CoV-2 infection. One caveat to these findings was the magnitude of the prophylactic efficacy observed. While both rAAV9 and rSIV.F/HN mediated NC0321 expression resulted in a significant reduction in S-LV infection, this inhibition was not total, and residual S-LV infection was still observed. Any consequences of this limitation remain to be elucidated in follow-on studies. Crucially, both rAAV9 and rSIV.F/HN vectors have been shown to provide life-long sustained IgG expression,^9^ and the unique rSIV.F/HN platform can also be effectively repeatedly administered^27 28^ should therapeutic antibody levels need to be boosted to improve efficacy or augmented with alternate mAbs to inhibit immune escape.

Interestingly, despite antibody expression from rAAV8 vectors being considered for treatment of a number of pathogens of importance,^29 30^ and rAAV8 vector being thought to mediate transgene expression more ubiquitously than other rAAV serotypes,^31^ we found relatively poor performance with rAAV8. A number of potential mitigating circumstances/explanations for these findings with rAAV8 can be postulated, such as unfavourable expression kinetics (though largely discounted by the speed of NC0321 appearance in the serum), or unfavourable biodistribution and antibody clearance (largely discounted by the accumulation of NC0321 in the ELF).

Despite the complexity of our experimental setting, mice did not show symptoms of stress (eg, weight loss) during the experiments (online supplemental figure 1F); and thus all the studies reported here were performed while imposing, at worst, only mild perturbations of the animals physiology—a significant refinement over traditional approaches to respiratory pathogen challenge/protection studies where humane endpoints are often used to minimise animal suffering. Importantly, studies assessing the degree of protection offered against more realistic (sub-saturating) infective S-LV doses and challenge with authentic SARS-CoV-2 will be incorporated in future work. Such studies may include evaluation of survival and immunological consequences.

Importantly, we showed that NC0321 can bind to different SARS-CoV-2 variants (figure 5D) including: the RBD mutation N501Y present in the Alpha (B.1.1.7) lineage SARS-CoV-2 strain widely circulating in the UK at the beginning of 2021^32^; the RBD with mutation Y453F is found in the mink-associated CoV-2 variant in the Netherlands. The binding and neutralising activity of NC0321 with other strains is currently being investigated, including the Beta lineage (B.1.351) first identified in South Africa (with key mutations N501Y+E484K+K417N) and Gamma lineage (P.1) first identified in the Brazilian population (with key mutations K417T+E484K+N501Y). We are also investigating whether the binding activity of NC0321 is compromised with the crucial E484K mutation. If not, NC0321 may be rapidly adapted in a COVIP setting for these newly emerging and more infectious SARS-CoV-2 clades. Notably, our approach is not limited to NC0321 antibody or to the use of a single mAb reagent. Indeed, we note that serum levels of NC0321 achieved following in vivo vector delivery were lower than can be achieved by optimisation of the IgG backbone sequences.^8^ Thus, enhanced derivatives of NC0321 or emerging mAbs with higher potency and more broadly neutralising activity^33 34^ could be readily assembled in rAAV9 or rSIV.F/HN vectors. Importantly, a cocktail strategy with multiple mAbs containing non-overlapping/non-competing antigen binding to Spike and other SARS-CoV-2 targets could be used to enhance the protection offered and prevent rapid mutational escape.^35^ For example, the Regeneron’s antibody cocktail Ronapreve (casirivimab and imdevimab) authorised by US Food and Drug Administration.^36^ Similarly, soluble receptor decoys engineered to efficiently neutralise SARS-CoV-2 could be incorporated into the vector cocktail to boost our SARS-CoV-2 inhibitory strategy.^37–39^

In conclusion, by using a versatile, humanised mouse model and SARS-COV-2 mimic, we evaluated a VIP strategy against COVID-19. An intranasal delivery route was simple to implement and the long duration of IgG expression observed benefited from the very slow turnover rate of lung cells. In murine studies, the lung tissue is easily accessible for localised vector administration via instillation. When translated to humans, this prophylactic approach could be delivered via nasal spray to provide protection against respiratory diseases in all recipients; these include but are not limited to vulnerable individuals who are unable to mount an effective immunological response to either SARS-CoV-2 infection or vaccination.

## Data Availability

All data relevant to the study are included in the article or uploaded as supplementary information.
