# Supplementary material for: Lung directed antibody gene transfer confers protection against SARS-CoV-2 infection
Source: Thorax. 2022 Feb 13:thoraxjnl-2021-217650. doi: 10.1136/thoraxjnl-2021-217650 (PMC8861887; doi:10.1136/thoraxjnl-2021-217650)

Supplementary methods

Virus vectors

Viral vector genome plasmids were engineered to include for rAAV8 a muscle-optimised CASI promoter [1], for rAAV9 the lung optimised hCEFI promoter [2], or for rSIV the hCEF derivative, lacking a chimeric intron [3]; all incorporated the Woodchuck Hepatitis Virus Post-transcriptional Regulatory Element (WPRE) to enhance expression [4], and a mirT-142-3p to improve immunologic tolerance [5]. Vectors used included eGFP, firefly luciferase or chimeric human IgG1 cDNAs (NC0321 anti-SARS-CoV-2 or T1-3B anti-influenza); IgG heavy and light chains were co-expressed in a single open reading frame (ORF) configuration [6]. For rAAV6.2 or rAAV9 hACE2 expression, a similar vector genome configuration with the CMV transgene promoter was used. The S-LV genome was a third-generation HIV vector including a CMV transgene promoter and an eGFP or firefly luciferase cDNA.

The production of rAAV, rSIV.F/HN and S-LV vectors was performed by co-transfection of human embryonic kidney (HEK) 293T cells with genome and packaging plasmids using (rAAV) Polyethylenimine (PEI, PolySciences) or (rSIV.F/HN and S-LV) PEIpro® (Polyplus). All AAV vectors included AAV2-based vector genomes. Alternate AAV serotypes were produced by use of AAV2 Rep and appropriate serotype Cap sequences. All rSIV vectors were pseudotyped with the F and HN proteins of Sendai virus as described previously [3]. The S-LV (rHIV.Spike.G614+Δ19aa.CMV) vector was pseudotyped with the SARS-CoV-2 Spike protein Wuhan sequence (GenBank accession: 43740568) modified by inclusion of a D614G mutation and removal of the 19 C-terminal amino acids. Vectors were purified by (rAAV) discontinuous Iodixanol gradient ultracentrifugation [7] or (rSIV.F/HN, S-LV) anion exchange chromatography and tangential flow filtration.

Physical titre (genome copies/mL: GC/mL) of rAAV vectors was determined by quantitative polymerase chain reaction (qPCR) analysis with primers and a probe against WPRE [8]. Functional titre of rSIV.F/HN vectors (transducing units/mL: TU/mL) was determined using the same primer/probes on DNA extracted following transduction of HEK293F cells with dilutions of vector preparations [9]. Physical titre (ng

p24) of S-LV particles was determined using a p24 immunoassay (SEK11695, Sino Biological).

Animal studies

All procedures involving laboratory mice were carried out in accordance with UK Home Office approved project and personal licenses under the terms of the Animals (Scientific Procedures) Act 1986 (ASPA 1986). Animals were arbitrarily assigned to study groups using an open-label randomised block approach. Overall, 134 animals were used (Fig 1: n=15, Fig 2: n=34, Fig 3: n=15, Fig 4/5: n =70).

Vector administration

Female BALB/c mice (5-8 week) were purchased from Envigo RMS UK. For all dosing procedures, mice were lightly anaesthetised by isoflurane. Where lung expression of hTMPRSS2 and/or hACE2 was required, cocktails of rAAV vectors were delivered by nasal instillation (I.N.) of a 100μL volume onto the nares via a single and continuous droplet [10]. Where mAb or control transgene expression was required, the relevant rAAV9 or rSIV.F/HN vector was included in the same cocktail. For intramuscular injection (I.M.) (AAV8), a 50 μL volume was injected via 22-gauge needle into the gastrocnemius muscle [9]. After the indicated period, specified animals were challenged with S-LV, which was also delivered via nasal instillation as described.

Immunohistochemistry and *In Situ Hybridization*

Left lung sections of BALB/c mice were isolated and fixed with 4% paraformaldehyde, embedded in sucrose 30% (w/v) at RT overnight, followed by injection of Optimal Cutting Temperature medium and 30% (w/v) sucrose mixture. The embedded left lung was cryo-sectioned at 7 μm thickness. For immunohistochemistry (IHC) analysis, sections from each sample was stained after antigen retrieval. Slides were washed, permeabilised, blocked, followed by the incubation in 20 μg/ml of primary antibody anti-eGFP antibody (ab6556, 1:1000, Abcam) overnight and subsequently with secondary antibody Goat Anti-Rabbit IgG H&L (Alexa Fluor 488, ab150077, at 1:500, Abcam). Sections were mounted under coverslips with DAPI pro-long antifade (P36935, Invitrogen™) before imaging using an EVOS FL2 fluorescence microscope with an 20X long working distance objective. For *In Situ Hybridization*, slides were

probed targeting the WPRE sequence (WPRE-O1, ACD, #450261) found in all AAV expression cassettes in this study, and/or the ATII cell marker Surfactant protein B (*Sftpb*-noX-human, ACD, #539421-C2) according to the RNAscope® manufacturer's instructions (323100-USM, ACD). Opal dyes 520 and 570 (#NEL741E001KT and #NEL744E001KT, 1:1500, Perkin Elmer) were used to visualise the *Sftpb* and WPRE signal, respectively.

In Vivo Luciferase Imaging

In vivo lung luciferase activity was determined following S-LV administration using IVIS spectrum imaging system (IVIS Lumina LT, Series III, PerkinElmer). This highly sensitive and linear method to determine lung transgene expression levels [11] can be achieved utilising recovery anaesthesia - allowing repeated measure in the same animal, allowing a substantial reduction in animal numbers over conventional luciferase activity measures that require lung tissue that can only be provided with a terminal procedure. Briefly, at the chosen time-points, *in vivo* lung luciferase activity was determined 10 minutes after mice were administered 100 µl 15 mg/ml D-luciferin (Xenogen Corporation Alameda) via the I.N method described for vector administration. Average bioluminescence (photons/sec/cm^2/sr) values are presented using a pseudo colour range to represent light intensity. Bioluminescence in each murine lung was measured within a standardised tissue area.

IgG expression profiling and binding activity

At indicated time points post vector delivery, serum and Broncho alveolar lavage fluid (BALF) was obtained [9]. Human IgG expression in serum and BALF was measured by ELISA (anti-Human IgG Fc domain, Bethyl Laboratories) according to the manufacturer's instructions. Urea levels were measured in serum and BALF (ab83362, Abcam). The concentrations of IgG levels in epithelial lining fluid (ELF) were determined by correcting for BALF sample dilution via normalizing found urea levels in serum and BALF [12].

Statistical analysis

Treatment group sizes were selected to achieve >0.8 power using G*Power 3.1.9.6 software [13]. Post-hoc statistical analysis was performed using Prism 8.4.3 (GraphPad

Software). Where possible, comparisons of multiple treatment groups were performed using one-way ANOVA followed by Dunnett's multiple comparisons test to a chosen comparator group or Tukey's comparison of all groups as appropriate; if necessary, data were log-normalised to assure adherence to the assumptions of ANOVA. Where the assumptions of one-way ANOVA were violated, the non-parametric Kruskal-Wallis test followed by Dunn's multiple comparisons test to a chosen comparator group was used. Where indicated, Area Under Curve (AUC) of time-course studies were computed from individual data, multiple comparisons of AUC between treatment groups were performed as described above. Errors were reported as the standard deviation of the mean (SD). In all cases, p value < 0.05 was considered statistically significant. In figures, ns, *, **, *** and **** indicate p-values of >0.05, <0.05, <0.01, <0.001 and <0.0001 respectively.

Supplementary Figure 1

A SDS-PAGE analysis reducing (left) and non-reducing (right) of produced monoclonal antibody. In reduced form, two bands were shown in 50 and 25 kDa and in non-reducing SDS-PAGE condition, only one band was seen in about 150 kDa indicating full length of IgG.

B Delivery of rAAV9 NC0321 and rSIV.F/HN.NC0321 to mouse lungs significantly inhibits S-LV infection. BALB/c mice (n=10/group) were treated with the indicated doses of rAAV9.hACE2, and rSIV.F/HN, rAAV9 or rAAV8 expressing either NC0321 or the T1-3B isotype control by the I.N. or I.M. route as indicated. 21 days later, mice were infected with 470 ng p24 of an S-LV expressing firefly luciferase. Representative *in vivo* bioluminescence images of mouse lungs 7 days post S-LV infection are shown for each treatment group. Bioluminescence values (photons/sec/cm^2/sr) are presented as a pseudocolor scale as indicated. Of note: one mouse in rAAV9.T1-3B group had very low bioluminescent signal, this was likely caused by a failure to deliver S-LV. This animal's data was included in the group analysis.

C Time-course of bioluminescence imaging data for the indicated treatment groups after infection with 470 ng p24. Symbols represent group mean±SD. The dotted line indicates the mean naïve background signal.

D Area Under Curve (AUC) of bioluminescence (photons/sec/cm2/sr x days) values for each animal receiving T1-3B was computed, symbols represent individual animals and group mean±SD (ANOVA, Dunnett's multiple comparison against the unlabelled treatment group; ns, and **** represent *p>0.05* and *<0.0001* respectively). The dotted line indicates the mean signal from naïve mice.

E Sera from animals receiving rAAV8.NC0321 inhibit S-LV.eGFP infection of hACE2+hTMPRSS2-expressing 293T cells (representative of n=2 independent infections).

F Weights of mice treated as described in **A** (n=10/group). Box and whisker plots represent the indicated treatment groups (inter-quartile range is shown as coloured vertical bars, group median is indicated with the horizontal line, whisker represent the 5-95% range).

Reference

1. Balazs AB, Chen J, Hong CM, et al. Antibody-based protection against HIV infection by vectored immunoprophylaxis. *Nature* 2011;481(7379):81-4. doi: 10.1038/nature10660 [published Online First: 2011/12/06]
2. Hyde SC, Pringle IA, Abdullah S, et al. CpG-free plasmids confer reduced inflammation and sustained pulmonary gene expression. *Nat Biotechnol* 2008;26(5):549-51. doi: 10.1038/nbt1399 [published Online First: 2008/04/29]
3. Alton EW, Beekman JM, Boyd AC, et al. Preparation for a first-in-man lentivirus trial in patients with cystic fibrosis. *Thorax* 2017;72(2):137-47. doi: 10.1136/thoraxjnl-2016-208406 [published Online First: 2016/11/16]
4. Zufferey R, Donello JE, Trono D, et al. Woodchuck hepatitis virus posttranscriptional regulatory element enhances expression of transgenes delivered by retroviral vectors. *J Virol* 1999;73(4):2886-92. doi: 10.1128/JVI.73.4.2886-2892.1999 [published Online First: 1999/03/12]
5. Annoni A, Brown BD, Cantore A, et al. In vivo delivery of a microRNA-regulated transgene induces antigen-specific regulatory T cells and promotes immunologic tolerance. *Blood* 2009;114(25):5152-61. doi: 10.1182/blood-2009-04-214569 [published Online First: 2009/10/02]
6. Balazs AB, Bloom JD, Hong CM, et al. Broad protection against influenza infection by vectored immunoprophylaxis in mice. *Nat Biotechnol* 2013;31(7):647-52. doi: 10.1038/nbt.2618 [published Online First: 2013/06/04]
7. Ojala DS, Amara DP, Schaffer DV. Adeno-associated virus vectors and neurological gene therapy. *Neuroscientist* 2015;21(1):84-98. doi: 10.1177/1073858414521870 [published Online First: 2014/02/22]
8. Meyer-Berg H, Zhou Yang L, Pilar de Lucas M, et al. Identification of AAV serotypes for lung gene therapy in human embryonic stem cell-derived lung organoids. *Stem Cell Res Ther* 2020;11(1):448. doi: 10.1186/s13287-020-01950-x [published Online First: 2020/10/25]
9. Tan TK, Gamlen TPE, Rijal P, et al. Lung-targeting lentiviral vector for passive immunisation against influenza. *Thorax* 2020;75(12):1112-15. doi: 10.1136/thoraxjnl-2020-214656 [published Online First: 2020/09/05]
10. Rose AC, Goddard CA, Colledge WH, et al. Optimisation of real-time quantitative RT-PCR for the evaluation of non-viral mediated gene transfer to the airways. *Gene Ther* 2002;9(19):1312-20. doi: 10.1038/sj.gt.3301792 [published Online First: 2002/09/12]
11. Griesenbach U, Meng C, Farley R, et al. In vivo imaging of gene transfer to the respiratory tract. *Biomaterials* 2008;29(10):1533-40. doi: 10.1016/j.biomaterials.2007.11.017 [published Online First: 2007/12/25]
12. Rennard SI, Basset G, Lecossier D, et al. Estimation of volume of epithelial lining fluid recovered by lavage using urea as marker of dilution. *J Appl Physiol (1985)* 1986;60(2):532-8. doi: 10.1152/jappl.1986.60.2.532 [published Online First: 1986/02/01]
13. Faul F, Erdfelder E, Lang AG, et al. G*Power 3: a flexible statistical power analysis program for the social, behavioral, and biomedical sciences. *Behav Res Methods* 2007;39(2):175-91. doi: 10.3758/bf03193146 [published Online First: 2007/08/19]

Supplementary Figure 1

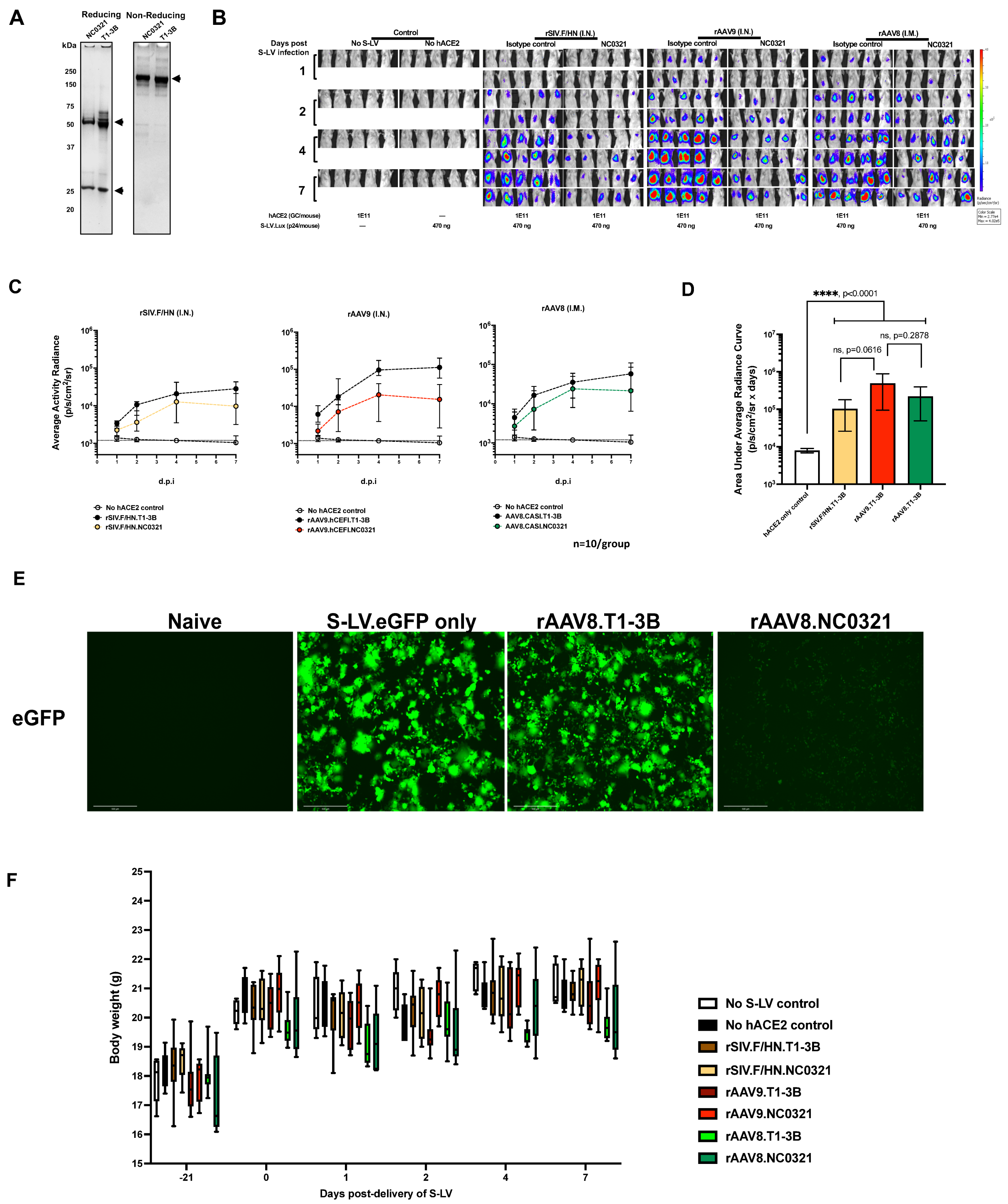

Supplement: Supplementary file 1 [file thoraxjnl-2021-217650supp001.pdf]
